# Factors associated with stillbirth in women with diabetes

**DOI:** 10.1007/s00125-019-4943-9

**Published:** 2019-07-29

**Authors:** Sharon T. Mackin, Scott M. Nelson, Sarah H. Wild, Helen M. Colhoun, Rachael Wood, Robert S. Lindsay, on behalf of the SDRN Epidemiology Group and Scottish Diabetes Group Pregnancy subgroup

**Affiliations:** 1 Institute of Cardiovascular and Medical Sciences, British Heart Foundation Glasgow Cardiovascular Research Centre, University of Glasgow, 126 University Place, Glasgow, G12 8TA UK; 2 School of Medicine, University of Glasgow, Glasgow, UK; 3 Usher Institute of Population Health Science and Informatics, University of Edinburgh, Edinburgh, UK; 4 Institute of Genetics and Molecular Medicine, University of Edinburgh, Edinburgh, UK; 5 ISD Scotland, Edinburgh, UK

**Keywords:** Birthweight, Epidemiology, Neonatal, Pregnancy, Stillbirth, Type 1 diabetes, Type 2 diabetes

## Abstract

**Aims/hypothesis:**

Stillbirth risk is increased in pregnancy complicated by diabetes. Fear of stillbirth has major influence on obstetric management, particularly timing of delivery. We analysed population-level data from Scotland to describe timing of stillbirths in women with diabetes and associated risk factors.

**Methods:**

A retrospective cohort of singleton deliveries to mothers with type 1 (*n* = 3778) and type 2 diabetes (*n* = 1614) from 1 April 1998 to 30 June 2016 was analysed using linked routine care datasets. Maternal and fetal characteristics, HbA_1c_ data and delivery timing were compared between stillborn and liveborn groups.

**Results:**

Stillbirth rates were 16.1 (95% CI 12.4, 20.8) and 22.9 (95% CI 16.4, 31.8) per 1000 births in women with type 1 (*n* = 61) and type 2 diabetes (*n* = 37), respectively. In women with type 1 diabetes, higher HbA_1c_ before pregnancy (OR 1.03 [95% CI 1.01, 1.04]; *p* = 0.0003) and in later pregnancy (OR 1.06 [95% CI 1.04, 1.08]; *p* < 0.0001) were associated with stillbirth, while in women with type 2 diabetes, higher maternal BMI (OR 1.07 [95% CI 1.01, 1.14]; *p* = 0.02) and pre-pregnancy HbA_1c_ (OR 1.02 [95% CI 1.00, 1.04]; *p* = 0.016) were associated with stillbirth. Risk was highest in infants with birthweights <10th centile (sixfold higher born to women with type 1 diabetes [*n* = 5 stillbirths, 67 livebirths]; threefold higher for women with type 2 diabetes [*n* = 4 stillbirths, 78 livebirths]) compared with those in the 10th–90th centile (*n* = 20 stillbirths, 1685 livebirths). Risk was twofold higher in infants with birthweights >95th centile born to women with type 2 diabetes (*n* = 15 stillbirths, 402 livebirths). A high proportion of stillborn infants were male among mothers with type 2 diabetes (81.1% vs 50.5% livebirths, *p* = 0.0002). A third of stillbirths occurred at term, with highest rates in the 38th week (7.0 [95% CI 3.7, 12.9] per 1000 ongoing pregnancies) among mothers with type 1 diabetes and in the 39th week (9.3 [95% CI 2.4, 29.2]) for type 2 diabetes.

**Conclusions/interpretation:**

Maternal blood glucose levels and BMI are important modifiable risk factors for stillbirth in diabetes. Babies at extremes of weight centiles are at most risk. Many stillbirths occur at term and could potentially be prevented by change in routine care and delivery policies.

**Electronic supplementary material:**

The online version of this article (10.1007/s00125-019-4943-9) contains peer-reviewed but unedited supplementary material, which is available to authorised users.

## Introduction

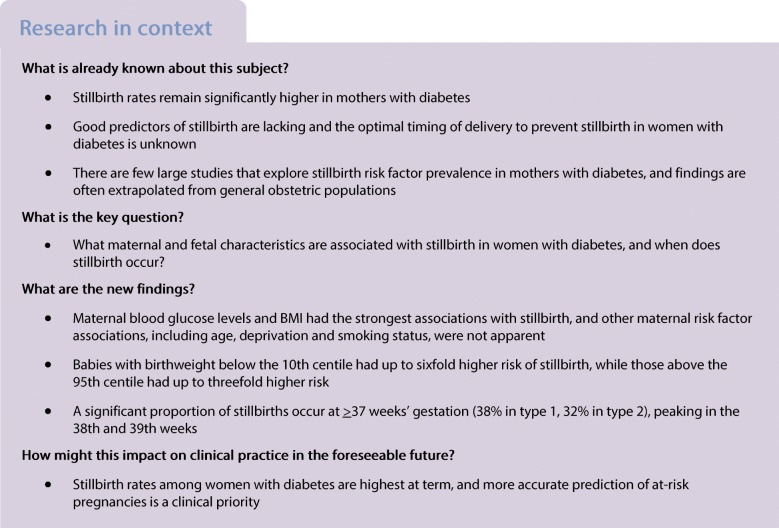



Mothers with pregestational diabetes are at 4–5-fold increased risk of stillbirth [1], with data from our and other populations showing no improvement in recent years [1, 2]. This contrasts with decreasing stillbirth rates seen in the general obstetric population [1]. Maternal obesity, advanced maternal age and smoking are important modifiable risk factors for stillbirth in the general obstetric population [3, 4]. Fetal growth is also important, with growth-restricted pregnancies having the highest risk [3]. Data on pregnancies complicated by diabetes are more limited. Suboptimal maternal blood glucose levels even at minimal levels, presence of microvascular complications and poor preparation for pregnancy are associated with stillbirth [5, 6]. Other traditional risk factors seen in the general obstetric population are less well documented in diabetes. Prevention of stillbirth underpins part of the clinical rationale for obstetric intervention in diabetes, particularly around timing of delivery. While we lack predictive models, presence of risk factors may guide obstetricians to earlier delivery, which is appropriate in many cases but associated with neonatal morbidity [7]. We therefore analysed national data from all deliveries to mothers with pregestational diabetes in Scotland over an 18 year period, to better define maternal and fetal characteristics associated with stillbirth. Timing of stillbirth was also analysed to identify potential for population-based strategies around routine delivery.

## Methods

### Data sources

As previously described [1], we linked data from maternity records in the Scottish Morbidity Record 02 (SMR02) database and the national diabetes database, Scottish Care Information-Diabetes (SCI-Diabetes). SMR02 contains clinical information on all obstetric inpatient episodes across Scotland including maternal and infant demographics, obstetric complications and delivery details. Quality assurance procedures have shown >90% completion and accuracy for data [8]. SCI-Diabetes contains patient demographics and clinical information on diabetes diagnosis, presence of complications and management. National data capture is excellent, with 99.5% of the Scottish population with diabetes included from 2004 onwards [9]. Diabetes diagnosis is entered onto SCI-Diabetes by individual clinical teams, and correlates with inpatient records in greater than 99% of cases [9]. For this study, type of diabetes was further refined by algorithm based on prescription history and age of diagnosis. Type 1 was reclassified as type 2 diabetes if there was more than 1 year without diabetes medications prescribed or treatment with oral hypoglycaemic agents only. Type 2 diabetes was reclassified as type 1 if diabetes was diagnosed under 30 years of age and initiated on insulin therapy within 1 year of diagnosis.

Episodes that resulted in the delivery of an infant at or beyond 24 weeks of gestation from 1 April 1998 to 30 June 2016 were identified from SMR02. Linkage with SCI-Diabetes identified mothers diagnosed with type 1 or type 2 diabetes predating delivery. Mothers with other diabetes diagnoses were excluded (*n* = 145). Analysis was restricted to singleton births only.

### Definitions

In keeping with the legal definition set out in the 1992 amendment of the Births and Deaths Registration (Scotland) Act 1965 [10], stillbirth was defined as the birth of an infant at or after 24 weeks of gestation, who at the time of delivery did not breathe or show signs of life. Gestational age has been calculated from ultrasound scanning in the first half of pregnancy in >95% of pregnancies since the 1990s [11].

Birthweight *z* score taking into account infant gestation at delivery, sex and parity was calculated using a reference population of all Scottish births between 1998 and 2003 [12]. Large for gestational age (LGA) describes infants whose corrected birthweights were above the 90th centile, while small for gestational age (SGA) included those below the 10th centile.

The Scottish Index of Multiple Deprivation (SIMD) 2012 score was used as a national standard measure of deprivation [13]. SIMD scores are calculated at small area (data zone) level based on multiple indicators of material deprivation, with women allocated to a score based on their postcode at time of delivery. Scores range from 0.89 to 89.89, with higher numbers reflecting residence in an area of higher material deprivation.

HbA_1c_ data are presented according to stages of pregnancy. The pre-pregnancy period was defined as 6 months preceding estimated conception date, and HbA_1c_ data were collected for the latest entry within that time. Conception date was calculated from gestational age and date of delivery. First trimester was defined as days 1–90 of pregnancy (up to week 12 + 6 days), trimester two as days 91–188 (week 13 + 0 days to week 26 + 6 days and trimester three from day 189 to delivery (week 27 onwards).

Mother’s BMI was available from routine diabetes clinic appointments (outwith pregnancy) and included as the last BMI within 6 months before pregnancy.

Healthcare provision in Scotland is divided into local health boards, defined by 14 distinct geographical areas [14]. We examined outcomes by health board of delivery and delivering hospital size, defined according to the mean number of deliveries of mothers with diabetes performed per annum (grouped as less than 5, 5 to 9, 10 to 19, 20 to 29, and 30 or greater deliveries). Very small units delivering under five women across the entire course of the study were not included. This represented 126 deliveries and under 1.8% of all data. Data for health boards are presented anonymised (A to K).

### Statistics

Data analysis was performed using Statistical Analysis Software (SAS) v.9.4 (Cary, NC, USA). Crude stillbirth rates are presented with a denominator of ongoing pregnancies at that gestational week. Risk factors and pregnancy outcomes are presented according to diabetes diagnosis for those in stillbirth and livebirth groups as means and SDs, or frequencies, as appropriate. As women may have had more than one pregnancy (range 1–8 with 67% of women having a single pregnancy and 94% one or two pregnancies), comparison between groups was assessed using a generalised mixed model with a term for the mother incorporated as a random effect. Differences in outcomes between health board and hospital unit size were analysed using logistic regression or general linear model with additional terms for year of delivery, deprivation score, maternal age, smoking and diabetes duration as appropriate. Due to low absolute numbers, effect of health board area of delivery and unit size on stillbirth rates was calculated from data combining type 1 and type 2 deliveries together, but including a term for type of diabetes. For analysis of stillbirth, two small island and one mainland health board were excluded due to low number of deliveries (<5 per annum). For analysis of the association of health board area and size with birthweight and gestational age at delivery, these were included. Statistical significance was assumed where *p* < 0.05.

The Caldicott Guardians of all Health Boards in Scotland and the Privacy Advisory Committee of the Information Services Division (ISD) of NHS Scotland approved database access and linkage. Ethical approvals were provided by the national multicentre research ethics committee. Data were accessed in a pseudonymised format.

## Results

There were 5621 pregnancies in mothers with diabetes, of which 229 were excluded for reasons including missing gestational age at delivery (*n* = 7), missing sex of infant (*n* = 3), delivery before 24 weeks of gestation (*n* = 5), diabetes diagnosis other than type 1 or type 2 (*n* = 145) or twin pregnancy (*n* = 74). Exclusions could have been in more than one category.

The remaining 5392 singleton babies (3778 offspring of mothers with type 1 diabetes, 1614 offspring of mothers with type 2 diabetes) were born to 3847 mothers (2582 mothers with type 1 diabetes, 1265 mothers with type 2 diabetes). Stillbirth rates were 16.1 per 1000 births (95% CI 12.4, 20.8) in type 1 diabetes (*n* = 61) and 22.9 per 1000 births (95% CI 16.4, 31.8) in type 2 diabetes (*n* = 37). Stillbirth rates did not change with time in type 1 diabetes. There was a very small reduction with time in type 2 diabetes (*p* = 0.02).

### Type 1 diabetes

Distributions of age, parity, smoking rates and deprivation scores were similar in mothers regardless of whether the pregnancy ended in stillbirth or livebirth (Table 1). Duration of diabetes was lower in the stillbirth group, as was pre-pregnancy BMI (Table 1).

**Table 1:** Maternal and neonatal factors associated with stillbirth according to infant vital status and type of diabetes

Factor	Type 1 diabetes (*n* = 3778 babies to 2582 mothers)		Type 2 diabetes (*n* = 1614 babies to 1265 mothers)	
	Stillbirth (*n* = 61)	Livebirth (*n* = 3717)	Stillbirth (*n* = 37)	Livebirth (*n* = 1577)
Maternal age at delivery, years	29.1 (5.3)	29.8 (5.7)	33.8 (6.0)	33.2 (5.6)
SIMD score	25.4 (16.4)	23.5 (17.1)	28.4 (19.1)	28.0 (18.1)
Duration of diabetes, years	11.4 (9.2)*	14.1 (8.4)	4.4 (4.0)	4.2 (4.1)
Nulliparous. %	55.7	50.0	16.7	31.3
Maternal smoking, %	25.5	20.4	30.3	21.0
Pre-pregnancy maternal BMI	24.8 (4.9)*	26.3 (4.9)	38.2 (6.4)*	33.9 (7.1)
Preconception HbA_1c_ below 53 mmol/mol (7%)^a^, %	7.9 (3 of 38)	19.6 (438 of 2236)	28.6 (4 of 14)	47.6 (380 of 799)
Male fetus, %	54.1	49.4	81.1***	50.5
Gestational age delivery, weeks	33.8 (4.1)***	36.6 (2.2)	33.7 (4.7) ***	37.2 (2.3)
Birthweight *z* score	1.38 (1.69)	1.37 (1.30)	1.08 (1.82)	0.83 (1.36)

Values are presented as percentage of group, or as mean (SD)

Missing values (*n*): Parity: 40 in type 1, 13 in type 2; maternal smoking: 294 in type 1, 128 in type 2; pre-pregnancy BMI: 1302 in type 1, 776 in type 2; preconception HbA_1c_: 1427 in type 1, 801 in type 2; birthweight *z* score: 50 in type 1, 13 in type 2

^a^Scottish Intercollegiate Guidelines Network (SIGN) recommended target HbA_1c_< 53 mmol/mol pre-pregnancy

*p < 0.05, ***p < 0.001 in generalised mixed models for stillbirth vs livebirth

**Fig. 1:**
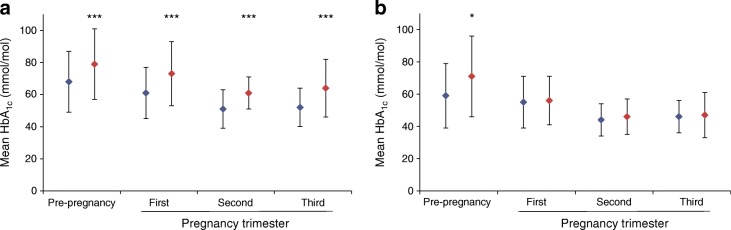
Mean HbA_1c_ according to stage of pregnancy and vital status of infant in women with (**a**) type 1 diabetes and (**b**) type 2 diabetes. Blue diamond, mean HbA_1c_ in livebirths; red diamond, mean HbA_1c_ in stillbirths. Error bars show SD. *p < 0.05, ***p < 0.01 for stillbirth vs livebirth in the same diabetes group at the same pregnancy stage using a generalised mixed model with mother as random effect. Number of available HbA_1c_ values: pre-pregnancy: 2313 (62%) livebirths and 38 (63%) stillbirths in women with type 1 diabetes; 796 (50%) livebirths and 17 (46%) stillbirths in women with type 2 diabetes; first trimester: 2156 (58%) livebirths and 30 (49%) stillbirths in women with type 1 diabetes; 785 (50%) livebirths and 15 (41%) stillbirths in women with type 2 diabetes; second trimester: 2119 (57%) livebirths and 29 (48%) stillbirths in women with type 1 diabetes; 775 (49%) livebirths and 16 (43%) stillbirths in women with type 2 diabetes; third trimester: 1933 (52%) livebirths and 27 (44%) stillbirths in women with type 1 diabetes; 782 (50%) livebirths and 11 (30%) stillbirths in women with type 2 diabetes

HbA_1c_ was recorded at each stage of pregnancy in 50–60% of cases (Fig. 1). Stillbirth rates were similar in women with an available pre-pregnancy HbA_1c_ measure to those without (1.6% both, *p* = 0.9), despite higher levels of deprivation and smoking (26.1% vs 17.0%) in those without a pre-pregnancy HbA_1c_ measurement (electronic supplementary material [ESM] Table 1). Overall, one in five women achieved pre-pregnancy glycaemic targets (<53 mmol/mol [7%]), with lower rates in the stillbirth group (Table 1). Mean pre-pregnancy HbA_1c_ was 11 mmol/mol (1%) higher in pregnancies ending in stillbirth (*p* = 0.0002). Glycaemic control improved from pre-pregnancy across successive trimesters in both stillborn and liveborn groups, but with the stillborn group maintaining a similar relative level of hyperglycaemia compared with the liveborn group at all points (Fig. 1).

**Fig. 2:**
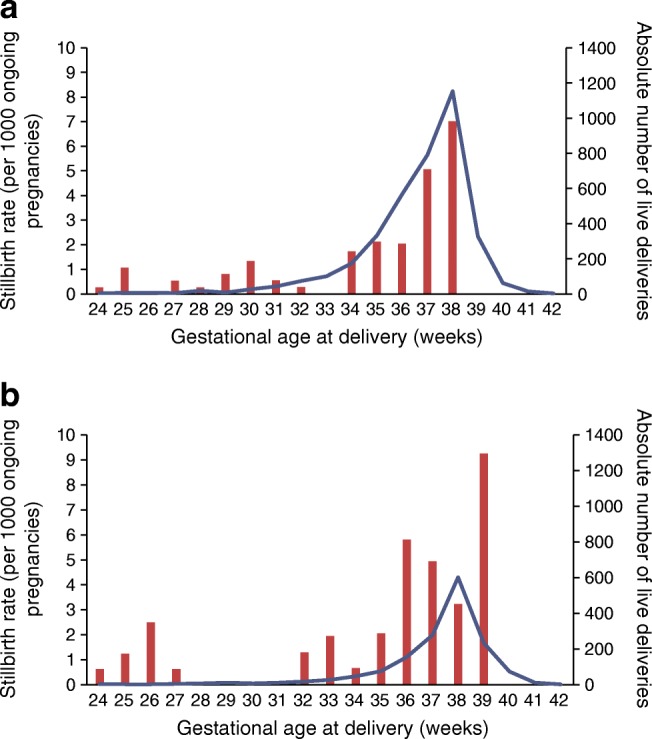
Stillbirth rate (per 1000 ongoing pregnancies) in women with (**a**) type 1 diabetes and (**b**) type 2 diabetes according to gestational age at delivery. Red bars, crude stillbirth rates (per 1000 ongoing pregnancies) on the *y*-axis; blue line, absolute number of live deliveries on the secondary *y*-axis

Stillborn infants were born 2.8 weeks earlier than liveborn infants (Table 1). Stillbirths occurred between weeks 24 and 38, with over a third (38%) at term (Fig. 2), compared with 63% of livebirths occurring at term. Stillbirth rates (expressed per week of pregnancy) were highest in the 37th and 38th weeks at 5.1 (95% CI 2.8, 9.1) and 7.0 (95% CI 3.7, 12.9) per 1000 ongoing pregnancies, respectively (Fig. 2). There were no stillbirths after 38 weeks, and only 11% of all deliveries occurred beyond this.

#### **HbA**_**1c**_**, birthweight and stillbirth risk**

Stillborn infants had similar corrected birthweights to liveborn infants, with birthweight *z* scores 1.38 and 1.37, respectively (Table 1). Over half of babies (52% combined live and stillborn) born to mothers with type 1 diabetes were LGA, of which the majority (78%) had birthweights above the 95th centile (ESM Table 2). Higher birthweight was related to higher HbA_1c_. Mean birthweight *z* scores for this combined group were 0.9 (0.12) SD higher than the reference population in the subgroup of women in the lowest quartiles for HbA_1c_ pre-pregnancy (<52 mmol/mol [6.9%]) and in the third trimester (<42 mmol/mol [6.0%]), increasing to 1.73 (0.09) SD for those in the highest quartiles (>76 mmol/mol [9.1%] pre-pregnancy and > 56 mmol/mol [7.3%] third trimester) (ESM Table 3). SGA infants had sixfold higher stillbirth rates than birthweight appropriate for gestational age infants (ESM Table 2). There was a significant non-linear relationship of birthweight *z* score and stillbirth (*p* = 0.002). When categorised as in ESM Table 2, there was a significant effect of birthweight category (*p* = 0.005) and SGA vs the reference category in particular.

### Type 2 diabetes

Mothers who had a stillbirth had higher mean pre-pregnancy BMI than mothers delivering a live infant (*p* = 0.01, Table 1) and with a non-significant trend to higher parity (*p* = 0.06). Stillborn infants were more likely to be male (*p* = 0.0007). Smoking, deprivation score and duration of diabetes were similar between groups (Table 1). HbA_1c_ was documented at each gestational period in approximately 50% of cases (Fig. 1). There was no difference in stillbirth rates in those with an available pre-pregnancy HbA_1c_ vs those without (2.1% vs 2.5%, respectively; *p* = 0.6). Almost half of women achieved pre-conceptual targets (<53 mmol/mol [7%]), with lower rates in the stillbirth group. There was no association between gestational blood glucose level and stillbirth (Fig. 1) but preconception HbA_1c_ was 12 mmol/mol (1.1%) higher in the stillbirth group (*p* = 0.01).

In type 2 diabetes, stillborn infants were delivered over 3 weeks earlier than liveborn infants (*p* < 0.0001) (Table 1). Stillbirth occurred in low frequency across all gestations from 24 weeks (Fig. 2), with 68% occurring preterm (Fig. 2) compared with 24% of livebirths occurring preterm. Stillbirth rates were highest in the 39th week at 9.3 (95% CI 2.4, 29.2) per 1000 ongoing pregnancies (Fig. 2).

#### HbA_1c_, birthweight and stillbirth risk

Mean birthweights were similar between live and stillborn infants (Table 1). Stillbirth odds were highest amongst infants with birthweights <10th centile (threefold) and > 95th centile (2.2-fold) (ESM Table 2). There was a significant non-linear relationship of birthweight *z* score and stillbirth (*p* = 0.007) and, when categorised as in ESM Table 2, there was a significant effect of birthweight category (*p* = 0.04), with suggestion of increased risk for both SGA and birthweight percentile >95th. Similar to type 1, babies (live and stillborn) born to mothers with HbA_1c_ in the lowest pre-pregnancy and third trimester quartiles were 0.39 (0.13) SD heavier than the reference population. This increased to 1.79 (0.26) SD for those with HbA_1c_ in the highest pre-pregnancy and third trimester quartiles (ESM Table 3).

### Risk factor effect estimation in stillbirth

In type 1 diabetes, univariate models suggested that shorter diabetes duration (OR 0.96 [95% CI 0.93, 0.99]; *p* = 0.01) and lower BMI (OR 0.92 [95% CI 0.86, 0.99]; *p* = 0.04) were weakly associated with stillbirth. There were significant associations between stillbirth and higher HbA_1c_ pre-pregnancy (OR 1.03 [95% CI 1.01, 1.04]; *p* = 0.0003) and in each trimester (OR 1.04 [95% CI 1.02, 1.05], 1.05 [95% CI 1.03, 1.07] and 1.06 [95% CI 1.04, 1.08] in first, second and third trimesters, respectively; all *p* < 0.0001). These effects remained in models adjusted for maternal age, SIMD and diabetes duration.

For type 2 diabetes, univariate models showed that higher BMI (OR 1.07 [95% CI 1.01, 1.14]; *p* = 0.02) and higher pre-pregnancy HbA_1c_ (OR 1.02 [95% CI 1.00, 1.04]; *p* = 0.016) were associated with stillbirth. In contrast to type 1 diabetes, HbA_1c_ later in pregnancy was not associated. These effects remained when adjusted for maternal age, SIMD and diabetes duration.

For type 1 diabetes, we further explored whether associations between HbA_1c_ and stillbirth varied by timing of HbA_1c_. Unsurprisingly, HbA_1c_ at various stages of pregnancy was highly correlated (ESM Table 4). In models including maternal age, diabetes duration, deprivation score, and pre-pregnancy and third trimester HbA_1c_ (*n* = 1382 with all variables), only third trimester HbA_1c_ remained significantly associated with stillbirth (OR 1.05 [95% CI 1.02, 1.08]; *p* = 0.0008). The effect of HbA_1c_ pre-pregnancy was attenuated (OR 1.02 [95% CI 0.99, 1.04]; *p* = 0.08). Third trimester HbA_1c_ did not have a significantly greater effect than pre-pregnancy HbA_1c_ (*p* = 0.12) on stillbirth but analysis was limited by low numbers of women with both HbA_1c_ measures.

For type 2 diabetes, the effect of BMI appeared independent of pre-pregnancy HbA_1c_, with both remaining associated with stillbirth (OR 1.09 [95% CI 1.02, 1.17]; *p* = 0.01 and 1.03 [95% CI 1.01, 1.05]; *p* = 0.006, respectively) in models including both factors, maternal age, diabetes duration and deprivation score (*n* = 594 with all values).

### Variation by region and unit size

There were no significant differences in stillbirth rates by health board area of delivery (*p* = 0.60) or by hospital unit size (*p* = 0.39). Regional differences in gestational age at delivery were observed for type 1 (range 33.4 ± 1.6 to 36.9 ± 0.2 weeks, *p* < 0.0001) and type 2 diabetes (range 36.4 ± 0.3 to 37.8 ± 0.2 weeks, *p* = 0.006). This appeared partly driven by earlier delivery in very small boards, but remained significant after exclusion of these in type 1 (range 36.1 ± 0.1 to 36.9 ± 0.2 weeks, *p* < 0.0001) and type 2 diabetes (range 36.5 ± 0.3 to 37.8 ± 0.2 weeks, *p* = 0.007) (ESM Fig. 1). Babies born at the largest units were delivered 4 days earlier than those delivering 10–19 mothers with diabetes per annum in type 1 (*p* = 0.002), but with no difference in type 2 diabetes. Mean birthweight *z* score in type 2 diabetes varied across health boards (*p* < 0.0001) (ESM Fig. 1), but not in type 1 diabetes (*p* = 0.05). Birthweight *z* score did not vary by hospital unit size in type 1 (*p* = 0.20) or type 2 diabetes (*p* = 0.15) in models including smoking (lower in smokers), year of delivery (small increase with time), maternal age at delivery (reduced with age), diabetes duration (increased with duration type 2 diabetes) and deprivation score (similar in type 2 diabetes, lower with higher SIMD in type 1 diabetes).

## Discussion

We have examined risk factors for stillbirth in a large population of mothers with diabetes. In keeping with other studies, we find that maternal blood glucose level is the key modifiable risk factor for adverse perinatal outcomes [6, 15, 16]. Women with type 1 diabetes who suffer a stillbirth have higher mean HbA_1c_ levels at all stages of pregnancy, although blood glucose level improves in both groups over the course of pregnancy. At the same time, there is large overlap in HbA_1c_ values between live and stillborn groups. The pattern of blood glucose levels in women with type 2 diabetes in relation to stillbirth appears somewhat different. Pre-pregnancy HbA_1c_ appears a more important predictor in type 2 diabetes, and unexpectedly there was no independent association in later pregnancy. The number of stillbirths in this group is small, however, and the findings should be interpreted with caution. An important clinical correlate is that pre-pregnancy counselling including glycaemic control may be particularly important to address in women with type 2 diabetes but uptake is generally lower than in type 1 diabetes [17]. Overall efforts to improve blood glucose levels before and during pregnancy remain central.

Despite lower blood glucose levels, mothers with type 2 diabetes had higher stillbirth rates than mothers with type 1 diabetes, in keeping with other reports [18, 19]. Maternal BMI was associated with stillbirth in this group. Maternal obesity is an independent risk factor for stillbirth, contributing to higher rates of preeclampsia, congenital anomalies and fetal overgrowth [20, 21]. Gestational weight gain would be of interest, with excessive gain conferring additional risk, but we lack data on late pregnancy weight to explore this further [22–24]. Other factors known to increase stillbirth include advancing maternal age [4], nulliparity, maternal smoking and social deprivation [3, 4]. While they were not formally statistically significant in their effects on stillbirth, we acknowledge their importance more widely in pregnancy outcomes.

The aetiology of stillbirth is still often unclear and we do not have specific information on causes in this cohort. In diabetes, at least some cases will reflect underlying congenital anomaly, the risk of which is associated with early pregnancy blood glucose levels [25]. However, in keeping with other studies, we expect that most stillbirths were unrelated to congenital anomalies, and instead caused by metabolic effects on fetal growth and placental function [26, 27].

Is it possible to predict those babies most at risk? Babies at the extremes of centiles for growth are known to have an increased risk of stillbirth [28]. In the general obstetric population, intrauterine growth restriction (IUGR) is the strongest indicator of stillbirth risk, increasing 4.0-fold if detected antenatally, and 8.0-fold if undetected [3]. In our data, absolute risk of stillbirth was highest in SGA infants, particularly in type 1 diabetes. There were very few babies born in this weight category (*n* = 78), with potential for overestimation of risk, but we think this is unlikely given similar increased risk seen in general obstetric populations [3]. In the general obstetric population, LGA infants are also at higher risk, particularly when weight is above the 95th centile [28]. This was demonstrated in women with type 2 diabetes but less apparent in type 1 diabetes. Routine obstetric care for women with diabetes recommends regular growth scans from 28 weeks of gestation to help identify at-risk pregnancies and allow earlier delivery where appropriate [29]. Given this likely pattern of obstetric intervention, similar rates of stillbirth in those at higher weights may represent some success of these policies. Birthweights of babies who were stillborn were similar to live births in type 1 and type 2 diabetes. We lack data on antenatal growth measures and have used birthweight as the only available measure to us to explore whether risk is predominantly in one growth group or another. Further, we accept that birthweight may underestimate fetal growth in the stillbirth group as it may have reduced or stopped prior to delivery. Fetal overgrowth relates to maternal hyperglycaemia in later pregnancy. However, it is notable that even in the subgroup of women with HbA_1c_ in the lowest quartiles pre-pregnancy (<52 mmol/mol [<6.9%]) and in the third trimester (<42 mmol/mol [6%]) birthweight remained considerably above that of the background population in both type 1 and 2 diabetes.

Optimal timing of delivery in diabetes remains controversial. In Scotland, routine delivery is recommended between 38 and 40 weeks, while the American College of Obstetricians and Gynecologists suggests delivery in the 39th week [29, 30], with individualised assessment for earlier delivery in those with additional risk factors. Recent National Institute for Health and Care Excellence (NICE) guidelines suggest earlier delivery during the 37th or 38th week, expedited for those with maternal and fetal complications [31]. In two prior series from the UK, RR of stillbirth in women with diabetes compared with the background population was increased at all stages of pregnancy [6, 32], but specifically at least fivefold at term [6, 32]. A third of the stillbirths in this study occurred at term, the majority of which would be expected to be antepartum rather than intrapartum. While it is facile to observe that earlier delivery might avoid these, it has to be balanced against increased risk of complications such as neonatal respiratory distress syndrome (RDS) [7]. We could speculate using these data that routine delivery of our population at 37 weeks would have resulted in approximately 142 cases of RDS with potential prevention of 22 stillbirths in type 1 diabetes (resulting in stillbirth rate 10.3 per 1000 births), and 72 cases of RDS with potential prevention of 12 stillbirths in type 2 diabetes (stillbirth rate 15.5 per 1000 births). However, the increased risk of neonatal morbidity would need to be more formally explored before recommendations for optimal timing of delivery are made, especially considering the very low representation of mothers with diabetes in current studies [7]. This balance will also be different in individuals achieving near normoglycaemia. For stillbirths occurring preterm, earlier delivery is less likely to prevent them until more accurate methods of predicting risk become available.

Others have suggested marked clinic-to-clinic variation in delivery of care to women with diabetes in pregnancy [33]. In Scotland, almost all care is organised in multidisciplinary clinics based in secondary (hospital) care. While units are of differing sizes, stillbirth rates do not appear significantly different. While accepting limitations of the power of our study, this is reassuring. There is slight variation in gestational age at delivery and birthweight, which may reflect migration of complex cases to larger centres for delivery, but may also indicate variation in obstetric practice relating to timing of delivery.

It was unexpected that male infants were much more likely to be stillborn in type 2 diabetes. Male fetuses are more vulnerable in utero, with risk moderately increased by 10% [34]. Yet our rates are fourfold higher in male infants than in females for type 2 diabetes (36.3 per 1000 male births vs 8.8 per 1000 female births). Male fetuses have higher metabolic demand in the later stages of pregnancy yet have smaller placentas than females, which may mean less compensatory reserve [35, 36]. Perhaps the combination of a vulnerable placenta and increased metabolic demand of the male infant explains this higher prevalence.

We have major strengths from robust, whole-population data that avoid selection bias but acknowledge shortcomings. First, these are observational data from a population in whom interventions, such as earlier delivery, will have occurred. Second, HbA_1c_ was only available in 50–60% of our population. After 2006–2008, this would have been from DCCT-aligned laboratories but there may have been greater variation between laboratories before this. HbA_1c_ may not always be measured in later pregnancy as it becomes a less robust correlate of maternal blood glucose level in second and third trimesters, and is not always routinely recommended [29, 37]. At the same time, our glycaemic results complement other studies [5, 27, 38, 39]. Third, we lack information across the whole of the study on important risk factors including microvascular complications. Nephropathy increases risk of IUGR and stillbirth [5, 40]. Methodologies for detecting microvascular renal disease (e.g. albuminuria) changed over the audit period in our population, limiting our ability to accurately report prevalence for the whole time course of the study. Information on BMI in early pregnancy and gestational weight gain is also lacking and would be of interest. Prescription information has only become robustly available in later years of the study. Folic acid supplementation is routinely recommended and would be of great interest, along with exposure to potentially teratogenic drugs. Finally, preeclampsia is a risk factor for IUGR and stillbirth [41–43]. Preeclampsia is known to be underreported in these routine data, and consistent with this it affects 6.1% of our type 1 diabetes and 4.5% of our type 2 diabetes cohorts, which is substantially lower than other population estimates [44].

In conclusion, maternal blood glucose level and BMI are the main modifiable risk factors associated with stillbirth in our population of women with diabetes. However, there is significant overlap in values between live and stillborn groups making it difficult to predict exactly which pregnancies will end in stillbirth. Achievement of near normoglycaemia remains key to reducing risk. Methods of supporting women to improve blood glucose levels in pregnancy along with programmes to optimise weight before pregnancy may help reduce stillbirth rates but are often challenging to implement successfully. Mortality risks are highest in infants born SGA, but large infants are also at increased risk. Stillbirth rate remains high at term in women with diabetes in our population, and until more accurate prediction of at-risk pregnancies is available, earlier delivery may be considered an attractive option.

## Electronic supplementary material

ESM (PDF 191 kb)

## Data Availability

Due to the confidential nature of clinical records, our data are not available in the public domain. SDRN Epidemiology Group have developed a series of algorithms refining pseudonymised datasets provisioned by ISD Scotland and can be contacted for collaboration.

## Abbreviations

IUGR: Intrauterine growth restriction
LGA: Large for gestational age
RDS: Respiratory distress syndrome
SCI-Diabetes: Scottish Care Information-Diabetes
SGA: Small for gestational age
SIMD: Scottish Index of Multiple Deprivation
SMR02: Scottish Morbidity Record 02
