# Supplementary material for: Factors associated with stillbirth in women with diabetes
Source: Diabetologia. 2019 Jul 29;62(10):1938–47. doi: 10.1007/s00125-019-4943-9 (PMC6731193; doi:10.1007/s00125-019-4943-9)
Supplement: Supplementary file 1 — (PDF 191 kb) [file 125_2019_4943_MOESM1_ESM.pdf]

ESM Table 1: Maternal and infant demographics according to availability of HbA_{1c} measurement pre-pregnancy and in each trimester

	Prepregnancy				2nd trimester				3rd trimester			
	Type 1 diabetes		Type 2 diabetes		Type 1 diabetes		Type 2 diabetes		Type 1 diabetes		Type 2 diabetes	
	HbA_{1c} available (n=2351)	No HbA_{1c} available (n=1427)	HbA_{1c} available (n=813)	No HbA_{1c} available (n=801)	HbA_{1c} available (n=2100)	No HbA_{1c} available (n=1678)	HbA_{1c} available (n=771)	No HbA_{1c} available (n=843)	HbA_{1c} available (n=1971)	No HbA_{1c} available (n=1807)	HbA_{1c} available (n=797)	No HbA_{1c} available (n=817)
**Maternal age at delivery, years (SD)**	30.1 (5.7)	29.1 (5.7)***	33.8 (5.5)	32.6 (5.6)***	29.8 (5.7)	29.7 (5.6)	33.4 (5.5)	33.0 (5.7)	29.8 (5.6)	29.7 (5.7)	33.5 (5.5)	32.9 (5.7)
**SIMD score (SD)**	22.6 (16.7)	25.0 (17.6)***	28.5 (18.0)	27.5 (18.3)	22.6 (16.0)	24.6 (18.2)***	27.8 (18.0)	28.1 (18.3)	22.6 (16.1)	24.6 (18.0) ***	27.7 (17.4)	28.3 (18.8)
**Duration of diabetes, years (SD)**	14.7 (8.4)	13.0 (8.4)***	4.8 (4.0)	3.6 (4.1)***	14.7 (8.4)	13.2 (8.4)***	4.5 (4.0)	3.9 (4.2)**	14.6 (8.5)	13.5 (8.3)***	4.4 (4.0)	4.0 (4.2)
**Nulliparous**	50.5% (n=1176)	49.3% (n=695)	30.8% (n=248)	31.2% (n=248)	50.9% (n=1057)	49.0% (n=814)	30.7% (n=235)	31.2% (n=261)	50.8% (n=986)	49.3% (n=885)	31.2% (n=248)	30.7% (n=248)
**Maternal Smoking**	17.0% (n=369)	26.1% (n=344)***	19.2% (n=143)	23.2% (n=172)	20.1% (n=398)	21.0% (n=315)	19.5% (n=140)	22.8% (n=175)	19.7% (n=366)	21.4% (n=347)	20.6% (n=152)	21.8% (n=163)
**Prepregnancy maternal BMI (SD)**	26.3 (4.7)	26.4 (5.5)	33.8 (7.1)	34.6 (7.1)	26.3 (5.0)	26.2 (4.7)	34.1 (7.1)	34.0 (7.2)	26.3 (5.0)	26.2 (4.7)	34.2 (7.3)	33.8 (6.9)
**Stillbirth rate**	16.2 (n=38)	16.1 (n=23)	20.9 (n=17)	25.0 (n=20)	13.3 (n=28)	19.7 (n=33)	19.5 (n=15)	26.1 (n=22)	13.7 (n=27)	18.8 (n=34)	13.8 (n=11)	31.8 (n=26)*
**Male fetus**	49.8% (n=1170)	48.9% (n=698)	51.2% (n=416)	51.3% (n=411)	49.6% (n=1042)	49.2% (n=826)	49.9% (n=385)	52.4% (n=442)	49.1% (n=967)	49.9% (n=901)	51.8% (n=413)	50.7% (n=414)
**Gestational age delivery, weeks (SD)**	36.6 (2.2)	36.6 (2.3)	36.9 (2.4)	37.3 (2.3)***	36.5 (2.1)	36.6 (2.4)	36.9 (2.4)	37.3 (2.4)***	36.8 (1.8)	36.3 (2.6)***	37.2 (1.8)	36.9 (2.9)**
**Birth weight Z-score (SD)**	1.43 (1.30)	1.28 (1.31)***	0.94 (1.41)	0.74 (1.32)**	1.38 (1.32)	1.36 (1.29)	0.94 (1.40)	0.75 (1.33)**	1.42 (1.28)	1.32 (1.33) *	0.91 (1.41)	0.77 (1.32)*

Values are presented as % of group, or mean ± SD, $^{*}p<0.05$, $^{**}p<0.01$, $^{***}p<0.001$ for stillbirth versus livebirth by, χ^2 or t-test

Scottish Index of Multiple Deprivation (SIMD) 2012 score is indicator of average material deprivation in area of residence at time of delivery. Scores range 0.89-89.89, with higher numbers reflecting residence in an area of higher material deprivation.

ESM Table 2: Stillbirth prevalence according to birth weight centile and diabetes diagnosis

	Birth weight centile				
**Type 1 diabetes (n=3728)**	**<10th (n=72)**	**10th to 90th (n=1705)**	**>90th (n=1951)**		**>95th (n=1527)**
**% stillbirth (n)**	6.9% (5)	1.2% (20)	1.7% (34)		1.7% (26)
**% livebirth (n)**	93.1% (67)	98.8% (1685)	98.3% (1917)		98.3% (1501)
**Odds Ratio stillbirth (95% confidence interval)**	6.3 (2.3-17.3)	Ref	1.5 (0.9-2.6)		1.5 (0.8-2.6)
**Type 2 diabetes (n=1600)**	**<10th (n=82)**	**10th to 90th (n=948)**	**>90th (n=570)**		**>95th (n=417)**
**% stillbirth (n)**	4.9% (4)	1.7% (16)	2.8% (16)		3.55% (15)
**% livebirth (n)**	95.1% (78)	98.3% (932)	97.2% (554)		96.45 (402)
**Odds Ratio stillbirth (95% confidence interval)**	3.0 (1.0-9.2)	Ref	1.7 (0.8-3.4)		2.2 (1.1-4.4)

Missing values (n): 50 in type 1, 14 in type 2

ESM Table 3: Mean birth weight Z-score according to combined pre-pregnancy and 3rd trimester HbA1c

	Type 1 diabetes				Type 2 diabetes			
	3rd trimester HbA_{1c} 1st quartile	3rd trimester HbA_{1c} 2nd quartile	3rd trimester HbA_{1c} 3rd quartile	3rd trimester HbA_{1c} 4th quartile	3rd trimester HbA_{1c} 1st quartile	3rd trimester HbA_{1c} 2nd quartile	3rd trimester HbA_{1c} 3rd quartile	3rd trimester HbA_{1c} 4th quartile
**Pre-pregnancy HbA_{1c} 1st quartile**	0.90 (0.12)	1.43 (0.14)	1.62 (0.19)	1.62 (0.30)	0.39 (0.13)	0.95 (0.17)	1.55 (0.29)	2.35 (0.43)
**Pre-pregnancy HbA_{1c} 2nd quartile**	0.73 (0.17)	1.32 (0.11)	1.54 (0.11)	1.70 (0.15)	0.38 (0.28)	0.74 (0.23)	1.51 (0.27)	1.93 (0.39)
**Pre-pregnancy HbA_{1c} 3rd quartile**	0.96 (0.23)	1.29 (0.14)	1.61 (0.11)	1.80 (0.12)	0.69 (0.34)	0.97 (0.32)	1.84 (0.39)	1.52 (0.36)
**Pre-pregnancy HbA_{1c} 4th quartile**	0.64 (0.27)	1.12 (0.19)	1.40 (0.13)	1.73 (0.09)	0.88 (0.36)	0.89 (0.31)	1.11 (0.29)	1.79 (0.26)

Quartiles defined by combined HbA_{1c} from type 1 and type 2 diabetes:

Pre-pregnancy quartile 1 <52 mmol/mol (6.9%); quartile 2 ≥52 mmol/mol (6.9%) and <63 mmol/mol (7.9%); quartile 3 ≥63 mmol/mol (7.9%) and <76 mmol/mol (9.1%); quartile 4 ≥ 76mmol/mol (9.1%)

3rd trimester quartile 1 <42 mmol/mol (6.0%); quartile 2 ≥42mmol/mol (6.0%) and < 49mmol/mol (6.6%); quartile 3 ≥49mmol/mol (6.6%) and <56 mmol/mol (7.3%); quartile 4 ≥ 56 mmol/mol (7.3%)

ESM table 4: HbA_{1c} value correlation at pre-pregnancy, 1st, 2nd and 3rd trimester in type 1 and type 2 diabetes

	Type 1 diabetes				Type 2 diabetes			
	**Pre-pregnancy HbA_{1c} (n=2351)**	**1st trimester HbA_{1c} (n=2251)**	**2nd trimester HbA_{1c} (n=2100)**	**3rd trimester HbA_{1c} (n=1971)**	**Pre-pregnancy HbA_{1c} (n=813)**	**1st trimester HbA_{1c} (n=828)**	**2nd trimester HbA_{1c} (n=771)**	**3rd trimester HbA_{1c} (n=797)**
**Pre-pregnancy HbA_{1c}**	1.000	0.687 (n=1658)****	0.493 (n=1512)****	0.466 (n=1382)****	1.000	0.733 (n=550)****	0.503 (n=495)****	0.478 (n=476)****
**1st trimester HbA_{1c}**	0.687 (n=1658)****	1.000	0.658 (n=1670)****	0.586 (n=1527)****	0.733 (n=550)****	1.000	0.604 (n=584)****	0.469 (n=551)****
**2nd trimester HbA_{1c}**	0.493 (n=1512)****	0.658 (n=1670)****	1.000	0.747 (n=1626)****	0.503 (n=495)****	0.604 (n=584)****	1.000	0.746 (n=606)****
**3rd trimester HbA_{1c}**	0.466 (n=1382)****	0.586 (n=1527)****	0.747 (n=1626)****	1.000	0.478 (n=476)****	0.470 (n=551)****	0.746 (n=606)****	1.000

**** $p<0.0001$

ESM Figure 1: Birth weight Z-score and gestational age at delivery according to health board area of delivery (1A: gestational age at delivery type 1 diabetes, 1B: gestational age at delivery type 2 diabetes; 1C: birth weight type 1 diabetes, 1D: birth weight type 2 diabetes).

Dots show mean value; error bars show standard deviation.

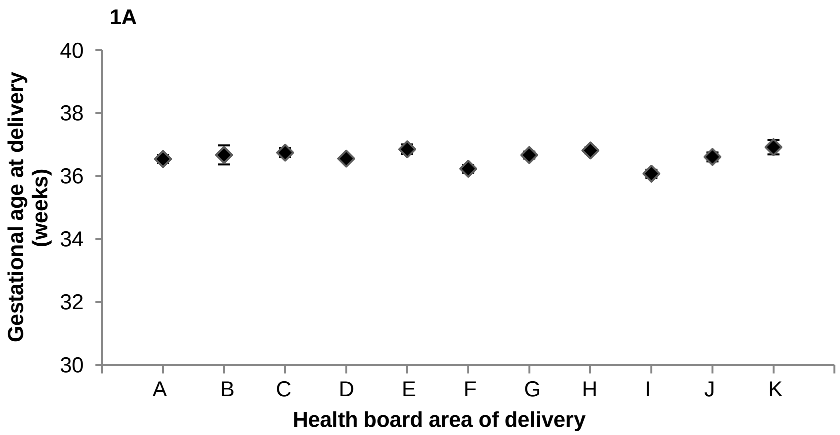

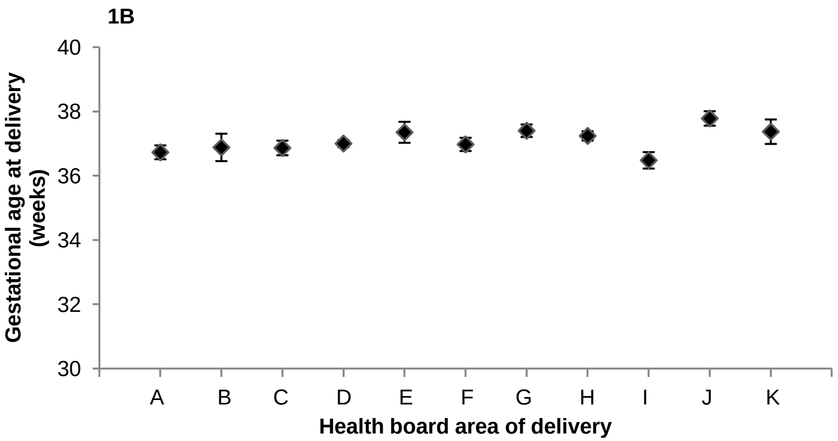

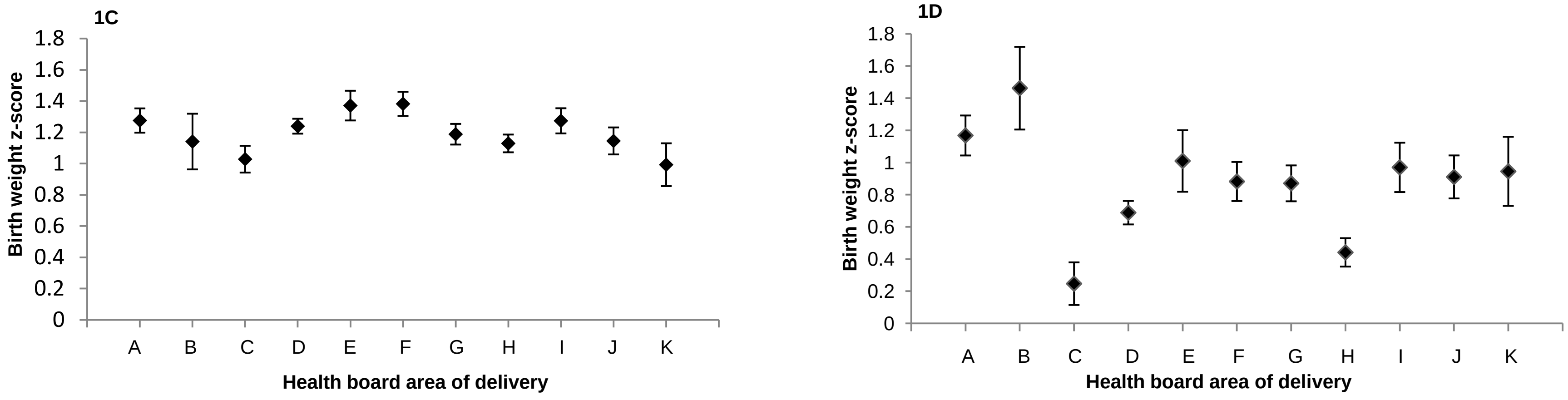

1C
Birth weight z-score
1.8
1.6
1.4
1.2
1
0.8
0.6
0.4
0.2
0
A B C D E F G H I J K
Health board area of delivery
1D
Birth weight z-score
1.8
1.6
1.4
1.2
1
0.8
0.6
0.4
0.2
0
A B C D E F G H I J K
Health board area of delivery
